# Persistent prelimbic cortex activity contributes to enhanced learned fear expression in females

**DOI:** 10.1101/lm.033514.113

**Published:** 2014-02

**Authors:** Georgina E. Fenton, Amelia K. Pollard, David M. Halliday, Rob Mason, Timothy W. Bredy, Carl W. Stevenson

**Affiliations:** 1 School of Biosciences, University of Nottingham, Sutton Bonington Campus, Loughborough LE12 5RD, United Kingdom; 2 School of Veterinary Medicine and Science, University of Nottingham, Sutton Bonington Campus, Loughborough LE12 5RD, United Kingdom; 3 Department of Electronics, University of York, Heslington, York YO10 5DD, United Kingdom; 4 School of Life Sciences, University of Nottingham, Queen’s Medical Centre, Nottingham NG7 2UH, United Kingdom; 5 Queensland Brain Institute, University of Queensland, Brisbane, Queensland 4072, Australia

## Abstract

Anxiety disorders, such as post-traumatic stress, are more prevalent in women and are characterized by impaired inhibition of learned fear and medial prefrontal cortex (mPFC) dysfunction. Here we examined sex differences in fear extinction and mPFC activity in rats. Females showed more learned fear expression during extinction and its recall, but not fear conditioning. They also showed more spontaneous fear recovery and more contextual fear before extinction and its recall. Moreover, enhanced learned fear expression in females was associated with sustained prelimbic (PL) cortex activity. These results suggest that sex differences in learned fear expression may involve persistent PL activation.

Women are at increased risk of developing fear-related anxiety disorders compared to men. For example, the prevalence of post-traumatic stress disorder (PTSD) is twice as high in women as in men (Lebron-Milad and Milad 2012). These disorders are characterized by impaired inhibition of learned fear (Milad et al. 2009a; Jovanovic et al. 2010) and a growing number of studies in humans and animals have shown sex differences in fear extinction (Milad et al. 2006; Baran et al. 2009, 2010; Glover et al. 2012; ter Horst et al. 2012; Baker-Andresen et al. 2013), the reduction in learned fear that occurs with repeated nonreinforced presentations of the conditioned stimulus (CS).

The neural circuitry mediating fear extinction is dysfunctional in PTSD. The medial prefrontal cortex (mPFC) is a heterogeneous area that plays a crucial role in this circuit through its involvement in learned fear and extinction processing. The dorsal anterior cingulate cortex (dACC) in humans and its rodent homolog, the prelimbic cortex (PL), are important for conditioned fear expression. In contrast, the human ventromedial prefrontal cortex (vmPFC) and the homologous infralimbic cortex (IL) in rodents are involved in fear suppression and extinction (Vidal-Gonzalez et al. 2006; Sierra-Mercado et al. 2011; Linnman et al. 2012). Importantly, PTSD is associated with dACC and vmPFC dysfunction (Milad et al. 2009a; Shin et al. 2009). Although a role for mPFC in mediating sex differences in fear extinction is emerging (Baran et al. 2010; Zeidan et al. 2011; Merz et al. 2012), the potential contribution of individual mPFC subregions remains unknown.

We examined sex differences in local field potential (LFP) activity in PL and IL during fear extinction in male and naturally cycling female Lister hooded rats (Harlan, UK). All experimental procedures were conducted with internal ethical approval and in accordance with the Animals (Scientific Procedures) Act 1986, UK. Electrodes (Teflon-coated stainless-steel wires, 50 μm diameter) were implanted into PL and IL (2.7 mm anterior and 0.5 mm lateral to bregma, 3.3 mm (PL) and 4.3 mm (IL) ventral to the brain surface) under isoflurane anesthesia. Rats received peri- and post-operative analgesia (buprenorphine and meloxicam) and were singly housed during recovery and behavioral testing, which started 10–14 d after surgery. On Day 0 rats were habituated to contexts A and B (15 min each). On Day 1 rats underwent tone habituation (five tones alone, 30 sec, 80 dB, 4 kHz, 2-min inter-trial interval [ITI]) followed by auditory fear conditioning (five tones co-terminating with footshock, 1 sec, 0.5 mA, 2 min ITI) in context A. On Days 2 and 15 rats underwent extinction training and recall testing (30 tones alone, 30-sec ITI), respectively, in context B (Fig. 1A). Freezing served as an index of conditioned fear and was scored manually.

**Figure 1.**
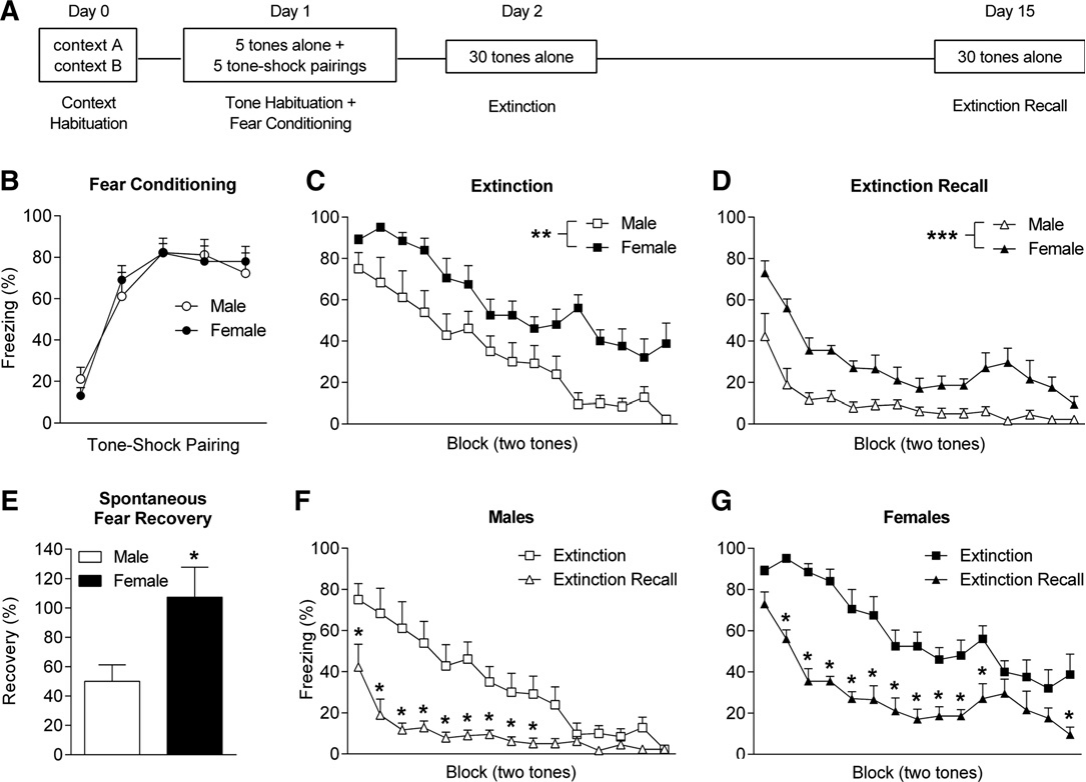
Females show enhanced learned fear expression during extinction and its recall. (*A*) Schematic representation of the behavioral testing paradigm used. (*B*) No sex differences in freezing were observed in response to tone–shock pairings during fear conditioning. Females showed increased tone-induced freezing during (*C*) extinction ([**] *P* < 0.01) and (*D*) extinction recall ([***] *P* < 0.001). (*E*) Females showed increased spontaneous recovery of fear (i.e., freezing at the start of extinction recall relative to the end of fear conditioning, [*] *P* < 0.05). Compared to extinction, tone-induced freezing was decreased during extinction recall in (*F*) males ([*] *P* < 0.05) and (*G*) females ([*] *P* < 0.05).

There were no differences between males (*n* = 9) and females (*n* = 10) in freezing during the presentation of tones alone (data not shown) or tone–shock pairings during fear conditioning (Fig. 1B). However, females showed significantly increased tone-induced freezing during extinction (two-way ANOVA, main effect of sex, *F*_(1,17)_ = 13.63, *P* < 0.01) (Fig. 1C) and extinction recall (two-way ANOVA, main effect of sex, *F*_(1,17)_ = 27.41, *P* < 0.001) (Fig. 1D). These results indicate that, although there were no sex differences in fear conditioning, females showed enhanced learned fear expression during extinction and its recall. Females also showed enhanced spontaneous recovery of fear, which is the return of conditioned fear over time after extinction (Quirk 2002). This was indicated by a significant increase in the percentage of fear recovered at the start of extinction recall relative to the end of fear conditioning (unpaired *t*-test, *t*_(17)_ = 2.39, *P* < 0.05) (Fig. 1E). However, freezing during extinction recall, compared to extinction, was significantly decreased in both males (two-way ANOVA, day × block interaction, *F*_(14,112)_ = 5.89, *P* < 0.0001; Bonferroni’s post-hoc test, Blocks 1–9, *P* < 0.05) (Fig. 1F) and females (two-way ANOVA, day × block interaction, *F*_(14,126)_ = 2.48, *P* < 0.001; Bonferroni’s post-hoc test, Blocks 2–11 and 15, *P* < 0.05) (Fig. 1G), indicating savings of extinction despite complete (i.e., ∼100%) spontaneous recovery of fear in females (Quirk 2002).

During behavioral testing LFP activity was recorded by connecting the implanted electrodes, via a headstage and a commutator, to a preamplifier linked to a Plexon Recorder system (Plexon Inc.). LFPs were band-pass filtered at 0.7–170 Hz and digitized at 1 kHz. Upon completion of the experiments rats were deeply anesthetized and current was passed through the electrodes. Rats were transcardially perfused with 0.9% saline followed by 4% paraformaldehyde/4% potassium ferrocyanide to mark the recording sites in PL and IL (Fig. 2A). LFP signals (Fig. 2B) were analyzed using multitaper spectral analysis, as previously described (Fenton et al. 2013). Spectral estimates of LFP activity during early and late extinction and extinction recall were generated by taking the average of the first two and last two tones during both sessions and then averaging across males or females. Confidence intervals (95%) were used to quantify statistically significant differences in PL and IL activity during early and late extinction and extinction recall separately in males and females. Given the recently established role of mPFC theta oscillations in mediating fear extinction (Lesting et al. 2011; Narayanan et al. 2011), we focused our analysis on this frequency band (4–12 Hz).

**Figure 2.**
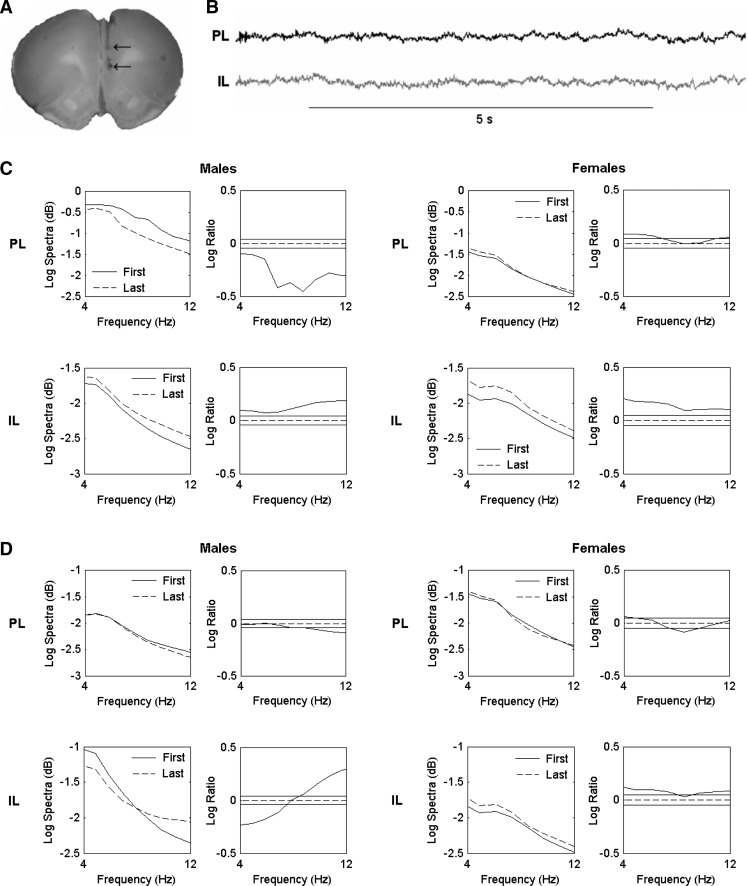
Females show sustained PL activation throughout extinction and its recall. (*A*) An example of electrode placements in PL and IL, indicated by the arrows. (*B*) Sample LFP traces recorded from PL and IL. (*C*) Pooled theta power spectra (*left*) and log ratio plots for pairwise comparisons of spectra (*right*) in PL (*top*) and IL (*bottom*) during the first and last tone blocks during extinction in males and females. The solid horizontal lines in the log ratio plots represent the upper and lower 95% confidence limits; positive log ratio values indicate increased power during the last compared to the first tone block, whereas negative values indicate decreased power during the last compared to the first tone block. In males, PL power decreased and IL power increased during extinction. In contrast, PL and IL power both increased during extinction in females. (*D*) Pooled theta power spectra (*left*) and log ratio plots (*right*) in PL (*top*) and IL (*bottom*) during the first and last tone blocks during extinction recall in males and females. In males, PL power decreased during extinction recall, albeit to a lesser extent compared to extinction. In IL, power shifted from lower to higher frequencies during extinction recall. In females, PL power showed little change and IL power increased during extinction recall.

In males, theta activity in PL showed a marked and significant decrease during late compared to early extinction (*P* < 0.05). Conversely, IL activity was significantly increased during late compared to early extinction (*P* < 0.05) (Fig. 2C). These results agree with evidence indicating the involvement of PL in fear memory expression and IL in the extinction and/or suppression of learned fear (Vidal-Gonzalez et al. 2006; Sierra-Mercado et al. 2011). The opposing patterns of theta activity, together with the low levels of theta synchronization observed between PL and IL during early and late extinction (coherence < 0.1) (data not shown), suggest that volume conduction of theta oscillations between these adjacent mPFC subregions was minimal. Females also showed significantly increased theta activity in IL during late compared to early extinction (*P* < 0.05). However, in contrast to males, PL activity showed a small but significant increase in the lower theta band during late compared to early extinction (*P* < 0.05) (Fig. 2C). We also observed sex differences in PL activity during extinction recall (Fig. 2D). PL activity showed a modest, albeit significant, decrease at higher theta frequencies during late compared to early extinction recall in males (*P* < 0.05). Males also showed a shift in IL activity from the lower (*P* < 0.05) to the higher (*P* < 0.05) theta range during late compared to early extinction recall. In females, there was little difference in theta activity in PL between late and early extinction recall, whereas theta activity in IL was significantly increased during late compared to early extinction recall (*P* < 0.05). These results suggest that females showed sustained PL activation during extinction and its recall, compared to males.

The mPFC forms part of the neural circuitry involved in the contextual regulation of learned fear expression and extinction (Orsini et al. 2011; Xu and Südhof 2013). To investigate the possibility that sex differences in learned fear expression and PL activity involved altered contextual fear regulation, we also examined contextual fear and mPFC activity before extinction and extinction recall. As expected, males showed low contextual fear in the 2-min period before the start of tone presentations during extinction and extinction recall. Females showed increased contextual fear before extinction and extinction recall compared to males (two-way ANOVA, main effect of sex, *F*_(1,17)_ = 8.70, *P* < 0.01) (Fig. 3A). In males, PL activity was significantly decreased in the lower theta band before extinction recall compared to before extinction (*P* < 0.05). However, in females, theta activity in PL was significantly increased before extinction recall compared to before extinction (*P* < 0.05) (Fig. 3B). Theta activity in IL was significantly increased before extinction recall compared to before extinction in males and, to a lesser extent, females (*P* < 0.05) (Fig. 3B).

**Figure 3.**
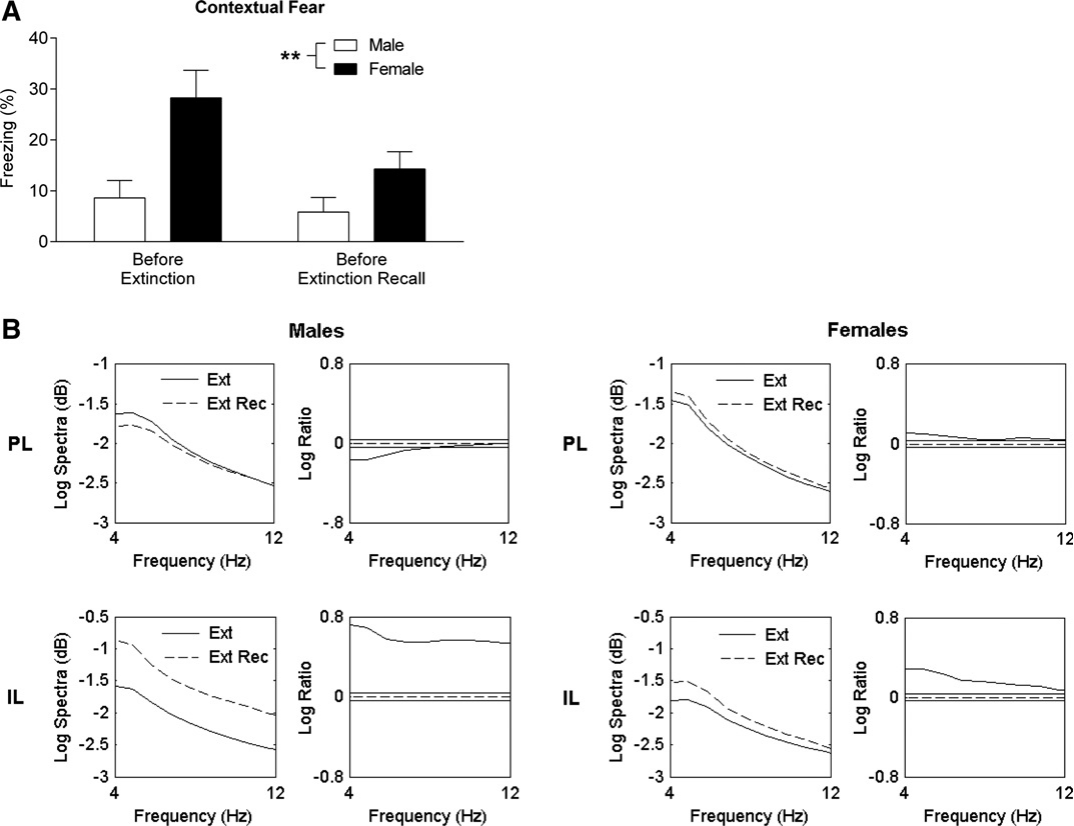
Females show enhanced contextual fear and PL activity before extinction recall. (*A*) Females showed increased freezing before tone presentations during extinction (*left*) and extinction recall (*right*) ([**] *P* < 0.01). (*B*) Pooled theta power spectra (*left*) and log ratio plots (*right*) in PL (*top*) and IL (*bottom*) before extinction (Ext) and before extinction recall (Ext Rec) in males and females. The solid horizontal lines in the log ratio plots represent the upper and lower 95% confidence limits; positive log ratio values indicate increased power before extinction recall compared to before extinction, whereas negative values indicate decreased power before extinction recall compared to before extinction. Males showed decreased PL power before extinction recall compared to before extinction. In contrast, females showed increased PL power before extinction recall compared to before extinction. Both males and females showed increased IL power before extinction recall compared to before extinction.

In this study we found no sex differences in fear conditioning. However, females showed enhanced learned fear expression throughout extinction the next day and extinction recall 2 wk later. Moreover, females showed enhanced spontaneous fear recovery, although both males and females demonstrated extinction savings. Females also showed enhanced contextual fear before extinction and its recall. These sex differences in learned fear expression were accompanied by altered PL, but not IL, activity. Males showed decreased PL and increased IL activity during extinction and extinction recall. Whereas females also showed increased IL activity during extinction and its recall, their PL activity increased during extinction and showed little change during extinction recall. Males and females also showed opposing patterns of PL activity during contextual fear expression before extinction and extinction recall. Males showed decreased PL activity in the “safe” context (i.e., before extinction recall) compared to the neutral context (i.e., before extinction), while females showed increased PL activity in the safe compared to the neutral context. Both males and females showed increased IL activity before extinction recall compared to before extinction. These results suggest that females show enhanced learned fear expression involving sustained PL activation.

Our finding of decreased PL and increased IL activity during extinction and its recall in males is in keeping with the known roles of PL and IL in mediating learned fear expression and its extinction, respectively. Electrical stimulation of PL enhances and reversible PL inactivation reduces learned fear expression (Vidal-Gonzalez et al. 2006; Sierra-Mercado et al. 2011). Single unit activity increases in some PL neurons during learned fear expression (Burgos-Robles et al. 2009) and our results suggest that fear memory expression is also represented by PL theta oscillations, which is a novel finding. In contrast to PL, reversible inactivation of IL impairs extinction (Sierra-Mercado et al. 2011) and single unit activity increases in IL neurons during extinction recall (Milad and Quirk 2002). Our finding of enhanced theta activity in IL during extinction agrees with recent evidence indicating a role for IL theta oscillations in extinction and its recall (Lesting et al. 2011; Narayanan et al. 2011). Our results showing increased IL activity before extinction recall compared to before extinction also support evidence indicating enhanced synaptic efficacy in IL after extinction (Herry and Garcia 2002). These findings add to evidence indicating the importance of theta oscillations in mediating learned fear and extinction processing by modulating neural excitability, plasticity, and synchronization in the fear memory circuit, in which mPFC plays a crucial role (Lesting et al. 2011).

During extinction recall we also observed decreased PL activity in males, although this decrease appeared to be less pronounced than during extinction. There was less of a difference in learned fear expression between early and late extinction recall, compared to early and late extinction, which could explain this discrepancy. A previous study showed complete spontaneous fear recovery 10–14 d after extinction in males (Quirk 2002). This finding differs from our results, which showed that fear recovered to ∼50% by 14 d after extinction. However, in that study all behavioral testing was conducted in the same context, while we conducted fear conditioning in a separate context to extinction and its recall. Extinction is well-known to be context-dependent (Orsini et al. 2011) and a study examining learned fear expression in the extinction context 7 d after extinction showed low conditioned fear during tone presentations (Herry and Garcia 2002), which agrees with our findings.

The lack of sex differences in fear conditioning reported here agrees with some, but not all, previous findings. Several studies have shown no sex differences in auditory fear conditioning (Maren et al. 1994; Markus and Zecevic 1997; Maes 2002; Baran et al. 2009; Baker-Andresen et al. 2013), although others have reported a deficit in females (Pryce et al. 1999; Kosten et al. 2005; Baran et al. 2010). Evidence also indicates that females show impaired contextual fear conditioning compared to males (Maren et al. 1994; Pryce et al. 1999; Gupta et al. 2001; Wiltgen et al. 2001; Chang et al. 2009). However, sex differences in aversive learning also depend on the conditioning paradigm used as females show enhanced eye-blink conditioning, fear-potentiated startle, and active avoidance compared to males (Dalla and Shors 2009).

At first glance our results broadly agree with evidence indicating resistance to fear extinction in females (Baran et al. 2009, 2010; Baker-Andresen et al. 2013). The sex difference observed in spontaneous fear recovery suggests that females show impaired extinction recall, although this finding could also be attributable to a deficit in the encoding of extinction. Interestingly, failure to recall extinction is associated with persistent PL activity (Burgos-Robles et al. 2009), as we found in females. However, females and males had similar patterns of IL activity before and during extinction and its recall. Females and males also both showed extinction savings, indicating the persistence of extinction memory (Quirk 2002). One intriguing possibility is that females show altered contextual regulation of, rather than resistance to, extinction. This could result in overgeneralization between the conditioning and extinction contexts, leading to enhanced expression of learned fear in the extinction context. Moreover, sustained PL activation related to learned fear expression in females may result from altered connectivity within the wider neural circuitry underlying the context dependency of extinction. The hippocampus is crucial for contextual processing and input from the ventral hippocampus (VH) to PL is involved in the contextual regulation of extinction (Orsini et al. 2011). Temporary VH inactivation increases PL activity and learned fear expression after extinction (Sotres-Bayon et al. 2012). This raises the possibility that females show alterations in hippocampal-mediated inhibition of mPFC function, which has been shown in PTSD (Milad et al. 2009a; Shin et al. 2009). Our finding of enhanced contextual fear before extinction and its recall in females, together with evidence indicating sex differences in contextual fear processing and hippocampal function (Maren et al. 1994; Gupta et al. 2001), supports this idea. Future studies could address this issue by examining sex differences in mPFC–hippocampus circuit function during contextual discrimination or fear renewal. This is the return of learned fear after extinction when the CS is presented outside of the extinction context and evidence indicates that fear renewal activates PL and hippocampus neurons (Knapska and Maren 2009). Although a previous study found no sex differences in the contextual regulation of fear extinction (Maes 2002), this issue is worth revisiting given the methodological differences between that study (e.g., strain, conditioned response measured, surgically naïve) and ours.

A limitation of this study is that we did not account for variations in the estrous cycle phase of the females. Previous studies in naturally cycling rats have shown that sex differences in fear extinction only emerged when the females were grouped according to their estradiol levels. Females with low levels showed impaired extinction recall compared to males and females with high levels (Milad et al. 2009b). Moreover, estrogen enhances the extinction of auditory and contextual fear conditioning (Chang et al. 2009; Zeidan et al. 2011). Interestingly, similar findings have been shown in women (Milad et al. 2010; Zeidan et al. 2011). Yet, despite not accounting for variations in the females’ estrous cycle phase, we still found sex differences in learned fear expression during extinction and its recall, which is broadly similar to the findings of others (Baran et al. 2009, 2010; Baker-Andresen et al. 2013). There is also evidence that estrogen can impair fear inhibition. Estrogen delayed the extinction of active avoidance in rats (Telegdy and Stark 1973). Naturally cycling women with high levels of endogenous estrogen showed impaired extinction recall compared to men and women with low estrogen levels (Milad et al. 2006). Estrogen also impaired fear inhibition in female rats by disrupting generalization between the CS and a second safety cue in a conditioned inhibition paradigm (Toufexis et al. 2007). Although the reasons for these discrepancies are unclear, they highlight the need for future studies to further address this important issue.

In summary, we found that females show enhanced learned fear expression associated with persistent PL activation, potentially due to altered contextual regulation of extinction. These results add to evidence indicating sex differences in cognition and mPFC function. Although the adaptive significance of these sex differences remains unclear, being able to respond to potential threats across different contexts may confer a survival advantage in females, but possibly at the expense of successfully adapting to changing stimulus contingencies in the environment (Baran et al. 2010).

## References

[FENTONLM033514C1] Baker-Andresen D, Flavell CR, Li X, Bredy TW 2013 Activation of BDNF signaling prevents the return of fear in female mice. Learn Mem 20: 237–240 23589089 10.1101/lm.029520.112

[FENTONLM033514C2] Baran SE, Armstrong CE, Niren DC, Hanna JJ, Conrad CD 2009 Chronic stress and sex differences on the recall of fear conditioning and extinction. Neurobiol Learn Mem 91: 323–332 19073269 10.1016/j.nlm.2008.11.005 PMC2673234

[FENTONLM033514C3] Baran SE, Armstrong CE, Niren DC, Conrad CD 2010 Prefrontal cortex lesions and sex differences in fear extinction and perseveration. Learn Mem 17: 267–278 20445082 10.1101/lm.1778010 PMC2862409

[FENTONLM033514C4] Burgos-Robles A, Vidal-Gonzalez I, Quirk GJ 2009 Sustained conditioned responses in prelimbic prefrontal neurons are correlated with fear expression and extinction failure. J Neurosci 29: 8474–8482 19571138 10.1523/JNEUROSCI.0378-09.2009 PMC2733220

[FENTONLM033514C5] Chang YJ, Yang CH, Liang YC, Yeh CM, Huang CC, Hsu KS 2009 Estrogen modulates sexually dimorphic contextual fear extinction in rats through estrogen receptor beta. Hippocampus 19: 1142–1150 19338017 10.1002/hipo.20581

[FENTONLM033514C6] Dalla C, Shors TJ 2009 Sex differences in learning processes of classical and operant conditioning. Physiol Behav 97: 229–238 19272397 10.1016/j.physbeh.2009.02.035 PMC2699937

[FENTONLM033514C7] Fenton GE, Spicer CH, Halliday DM, Mason R, Stevenson CW 2013 Basolateral amygdala activity during the retrieval of associative learning under anesthesia. Neuroscience 13: 146–156 23295986 10.1016/j.neuroscience.2012.12.039

[FENTONLM033514C8] Glover EM, Jovanovic T, Mercer KB, Kerley K, Bradley B, Ressler KJ, Norrholm SD 2012 Estrogen levels are associated with extinction deficits in women with posttraumatic stress disorder. Biol Psychiatry 72: 19–24 22502987 10.1016/j.biopsych.2012.02.031 PMC3675159

[FENTONLM033514C9] Gupta RR, Sen S, Diepenhorst LL, Rudick CN, Maren S 2001 Estrogen modulates sexually dimorphic contextual fear conditioning and hippocampal long-term potentiation (LTP) in rats. Brain Res 888: 356–365 11150498 10.1016/s0006-8993(00)03116-4

[FENTONLM033514C10] Herry C, Garcia R 2002 Prefrontal cortex long-term potentiation, but not long-term depression, is associated with the maintenance of extinction of learned fear in mice. J Neurosci 22: 577–583 11784805 10.1523/JNEUROSCI.22-02-00577.2002 PMC6758664

[FENTONLM033514C11] Jovanovic T, Norrholm SD, Blanding NQ, Davis M, Duncan E, Bradley B, Ressler KJ 2010 Impaired fear inhibition is a biomarker of PTSD but not depression. Depress Anxiety 27: 244–251 20143428 10.1002/da.20663 PMC2841213

[FENTONLM033514C39] Knapska E, Maren S 2009 Reciprocal patterns of c-Fos expression in the medial prefrontal cortex and amygdala after extinction and renewal of conditioned fear. Learn Mem 16: 486–493 19633138 10.1101/lm.1463909 PMC2726014

[FENTONLM033514C12] Kosten TA, Miserendino MJ, Bombace JC, Lee HJ, Kim JJ 2005 Sex-selective effects of neonatal isolation on fear conditioning and foot shock sensitivity. Behav Brain Res 157: 235–244 15639174 10.1016/j.bbr.2004.07.001

[FENTONLM033514C13] Lebron-Milad K, Milad MR 2012 Sex differences, gonadal hormones and the fear extinction network: Implications for anxiety disorders. Biol Mood Anxiety Disord 2: 3 22738383 10.1186/2045-5380-2-3 PMC3384233

[FENTONLM033514C14] Lesting J, Narayanan RT, Kluge C, Sangha S, Seidenbecher T, Pape HC 2011 Patterns of coupled theta activity in amygdala–hippocampal–prefrontal cortical circuits during fear extinction. PLoS One 6: e21714 21738775 10.1371/journal.pone.0021714 PMC3125298

[FENTONLM033514C15] Linnman C, Zeidan MA, Furtak SC, Pitman RK, Quirk GJ, Milad MR 2012 Resting amygdala and medial prefrontal metabolism predicts functional activation of the fear extinction circuit. Am J Psychiatry 169: 415–423 22318762 10.1176/appi.ajp.2011.10121780 PMC4080711

[FENTONLM033514C16] Maes JH 2002 No sex difference in contextual control over the expression of latent inhibition and extinction in Pavlovian fear conditioning in rats. Neurobiol Learn Mem 78: 258–278 12431417 10.1006/nlme.2002.4058

[FENTONLM033514C17] Maren S, De Oca B, Fanselow MS 1994 Sex differences in hippocampal long-term potentiation (LTP) and Pavlovian fear conditioning in rats: Positive correlation between LTP and contextual learning. Brain Res 661: 25–34 7834376 10.1016/0006-8993(94)91176-2

[FENTONLM033514C18] Markus EJ, Zecevic M 1997 Sex differences and estrous cycle changes in hippocampus-dependent fear conditioning. Psychobiology 25: 246–252

[FENTONLM033514C19] Merz CJ, Tabbert K, Schweckendiek J, Klucken T, Vaitl D, Stark R, Wolf OT 2012 Neuronal correlates of extinction learning are modulated by sex hormones. Soc Cogn Affect Neurosci 7: 819–830 21990419 10.1093/scan/nsr063 PMC3475362

[FENTONLM033514C20] Milad MR, Quirk GJ 2002 Neurons in medial prefrontal cortex signal memory for fear extinction. Nature 420: 70–74 12422216 10.1038/nature01138

[FENTONLM033514C21] Milad MR, Goldstein JM, Orr SP, Wedig MM, Klibanski A, Pitman RK, Rauch SL 2006 Fear conditioning and extinction: Influence of sex and menstrual cycle in healthy humans. Behav Neurosci 120: 1196–1203 17201462 10.1037/0735-7044.120.5.1196

[FENTONLM033514C22] Milad MR, Pitman RK, Ellis CB, Gold AL, Shin LM, Lasko NB, Zeidan MA, Handwerger K, Orr SP, Rauch SL 2009a Neurobiological basis of failure to recall extinction memory in posttraumatic stress disorder. Biol Psychiatry 66: 1075–1082 19748076 10.1016/j.biopsych.2009.06.026 PMC2787650

[FENTONLM033514C23] Milad MR, Igoe SA, Lebron-Milad K, Novales JE 2009b Estrous cycle phase and gonadal hormones influence conditioned fear extinction. Neuroscience 164: 887–895 19761818 10.1016/j.neuroscience.2009.09.011 PMC2783784

[FENTONLM033514C24] Milad MR, Zeidan MA, Contero A, Pitman RK, Klibanski A, Rauch SL, Goldstein JM 2010 The influence of gonadal hormones on conditioned fear extinction in healthy humans. Neuroscience 168: 652–658 20412837 10.1016/j.neuroscience.2010.04.030 PMC2881679

[FENTONLM033514C25] Narayanan V, Heiming RS, Jansen F, Lesting J, Sachser N, Pape HC, Seidenbecher T 2011 Social defeat: Impact on fear extinction and amygdala-prefrontal cortical theta synchrony in 5-HTT deficient mice. PLoS One 6: e22600 21818344 10.1371/journal.pone.0022600 PMC3144906

[FENTONLM033514C26] Orsini CA, Kim JH, Knapska E, Maren S 2011 Hippocampal and prefrontal projections to the basal amygdala mediate contextual regulation of fear after extinction. J Neurosci 31: 17269–17277 22114293 10.1523/JNEUROSCI.4095-11.2011 PMC3241946

[FENTONLM033514C27] Pryce CR, Lehmann J, Feldon J 1999 Effect of sex on fear conditioning is similar for context and discrete CS in Wistar, Lewis and Fischer rat strains. Pharmacol Biochem Behav 64: 753–759 10593198 10.1016/s0091-3057(99)00147-1

[FENTONLM033514C28] Quirk GJ 2002 Memory for extinction of conditioned fear is long-lasting and persists following spontaneous recovery. Learn Mem 9: 402–407 12464700 10.1101/lm.49602 PMC187587

[FENTONLM033514C29] Shin LM, Lasko NB, Macklin ML, Karpf RD, Milad MR, Orr SP, Goetz JM, Fischman AJ, Rauch SL, Pitman RK 2009 Resting metabolic activity in the cingulate cortex and vulnerability to posttraumatic stress disorder. Arch Gen Psychiatry 66: 1099–1107 19805700 10.1001/archgenpsychiatry.2009.138 PMC3752096

[FENTONLM033514C30] Sierra-Mercado D, Padilla-Coreano N, Quirk GJ 2011 Dissociable roles of prelimbic and infralimbic cortices, ventral hippocampus, and basolateral amygdala in the expression and extinction of conditioned fear. Neuropsychopharmacology 36: 529–538 20962768 10.1038/npp.2010.184 PMC3005957

[FENTONLM033514C31] Sotres-Bayon F, Sierra-Mercado D, Pardilla-Delgado E, Quirk GJ 2012 Gating of fear in prelimbic cortex by hippocampal and amygdala inputs. Neuron 76: 804–812 23177964 10.1016/j.neuron.2012.09.028 PMC3508462

[FENTONLM033514C32] Telegdy G, Stark A 1973 Effect of sexual steroids and androgen sterilization on avoidance and exploratory behaviour in the rat. Acta Physiol Acad Sci Hung 43: 55–63 4770671

[FENTONLM033514C33] ter Horst JP, Carobrez AP, van der Mark MH, de Kloet ER, Oitzl MS 2012 Sex differences in fear memory and extinction of mice with forebrain-specific disruption of the mineralocorticoid receptor. Eur J Neurosci 36: 3096–3102 22831399 10.1111/j.1460-9568.2012.08237.x

[FENTONLM033514C34] Toufexis DJ, Myers KM, Bowser ME, Davis M 2007 Estrogen disrupts the inhibition of fear in female rats, possibly through the antagonistic effects of estrogen receptor α (ERα) and ERβ. J Neurosci 27: 9729–9735 17804633 10.1523/JNEUROSCI.2529-07.2007 PMC6672956

[FENTONLM033514C35] Vidal-Gonzalez I, Vidal-Gonzalez B, Rauch SL, Quirk GJ 2006 Microstimulation reveals opposing influences of prelimbic and infralimbic cortex on the expression of conditioned fear. Learn Mem 13: 728–733 17142302 10.1101/lm.306106 PMC1783626

[FENTONLM033514C36] Wiltgen BJ, Sanders MJ, Behne NS, Fanselow MS 2001 Sex differences, context preexposure, and the immediate shock deficit in Pavlovian context conditioning with mice. Behav Neurosci 115: 26–32 11256449 10.1037/0735-7044.115.1.26

[FENTONLM033514C37] Xu W, Südhof TC 2013 A neural circuit for memory specificity and generalization. Science 339: 1290–1295 23493706 10.1126/science.1229534 PMC3651700

[FENTONLM033514C38] Zeidan MA, Igoe SA, Linnman C, Vitalo A, Levine JB, Klibanski A, Goldstein JM, Milad MR 2011 Estradiol modulates medial prefrontal cortex and amygdala activity during fear extinction in women and female rats. Biol Psychiatry 70: 920–927 21762880 10.1016/j.biopsych.2011.05.016 PMC3197763

